# Implementing texting programs in the P.O.W.E.R. (preventing obesity with eating right) medical group visit for weight loss

**DOI:** 10.1002/osp4.513

**Published:** 2021-05-04

**Authors:** Perla Saldivar, Valerie Mira, Petra Duran, Christina Moldovan, Georgina Ang, Nina Parikh, Martin L. Lee, Theodore C. Friedman

**Affiliations:** ^1^ Division of Endocrinology, Metabolism, and Molecular Medicine, Department of Internal Medicine Charles R. Drew University of Medicine and Science Los Angeles California USA; ^2^ CareMessage San Francisco California USA; ^3^ Martin Luther King Jr. Outpatient Center Los Angeles California USA

**Keywords:** exercise, group visit, HbA1c, medically underserved, obesity, texting, weight loss

## Abstract

**Background:**

The effect of incorporating mobile technology to support participants’ lifestyle change and weight loss in medical group visits has not been well studied in a safety‐net setting.

**Rationale and Design:**

Thus, the rationale of the current study was to examine the effect of text messaging in a medical group visit, and test the effect of two texting programs (12 weeks and 20 weeks), compared to those who did not receive text‐messaging in the Preventing Obesity With Eating Right (POWER) group visit program. The primary outcome was weight loss.

**Results:**

We found that those enrolled in the 20‐week and 12‐week texting programs attended more group visit sessions than those enrolled in the POWER group only (*p* < 0.001). Both POWER and POWER + 20‐week texting groups had a significant reduction in weight at their final group visit compared to their baseline (POWER, 114 ± 27 kg vs. 112 ± 26 kg, *p* < 0.001; POWER + 20‐week texting, 111 ± 28 kg vs. 109 ± 28 kg, *p* < 0.01), but not the 12‐week texting group (114 ± 29 kg vs. 113 ± 29 kg, *p* = 0.22), with no differences between the groups. The number of group visits was correlated with a decrease in weight (*r_s_* = 0.12, *p* < 0.05).

**Conclusion:**

In conclusion, text messaging programs led to more attendance in the medical group visits, but not greater weight loss or reduction in HbA1c than the POWER group obesity program alone. Further studies are needed to maximize the beneficial effects of texting programs in medical group visits in underserved minority populations.

## 1 INTRODUCTION

Obesity (a body mass index (BMI) ≥ 30 kg/m^2^) has become a major public health concern worldwide in the last 40 years. In the United States, obesity contributes to 100,000–400,000 excess deaths per year and is attributed to $117 billion dollars in medical expenditure.^1^, ^2^ Obesity poses a significant health disparity, largely affecting socioeconomically disadvantaged individuals which are disproportionately members of racial and ethnic minority groups.^3^ The burden of obesity in socioeconomically disadvantaged communities calls for innovative efforts that are both cost‐effective and sustainable. A study assessing weight loss maintenance over seven years found that weight‐loss maintainers used more behavioral strategies to control fat intake and more strenuous physical activity than weight‐regainers.^4^ These strategies include self‐monitoring, goal setting, eating habit modifications, and health behavior reinforcement.^5^ Two approaches that have been shown to significantly improve behavior change in chronic conditions are the medical group visit model and the use of mobile technology.^6^

Medical group visits, also known as “cooperative health care clinics,” “shared medical appointments,” or “group medical visits,” deliver care to patients with similar conditions in a group setting (8–30 individuals).^7^, ^8^ The model uses a multi‐disciplinary team that takes an educational approach to teach patients effective self‐management strategies in addition to providing individual medical attention.^9^ Medical group visit models benefit from both group therapy and the physician‐patient relationship. Studies demonstrate that medical group visits improve efficiency in healthcare delivery, patient satisfaction, and use of preventative services, while decreasing emergency clinical visits.^10^, ^11^, ^12^ Further, a study with low‐income women managing chronic conditions, largely of Hispanic descent, found that group medical visits increased personalized attention (77%), self‐care education (69%), and access to medication refills (69%), and significantly decreased urgent care visits during the 9‐month intervention compared to the 9 months prior to the intervention (*p* < 0.05).^13^

In the treatment of obesity, medical group visits have shown to lead to significant weight loss in developmentally delayed adults.^14^ Another study in a pediatric population who attended a medical group visit at least twice in a 3‐year period revealed improvement in BMI, stress, and healthy behaviors such as exercise and sleep, while decreasing unhealthy habits including high sugar beverage consumption, fast food intake, and television viewing time.^14^, ^15^ Despite the clear benefits of medical group visits, this care model poses potential limitations including logistical barriers such as transportation, missing appointments, family obligations, and difficulty relating to or supporting other group members from different racial/ethnic backgrounds.^13^

Innovative approaches such as incorporating mobile technology in medical group visit settings can address some of these logistical barriers, while continuing the behavior interventions outside of the visits. Mobile phones are used in a variety of domains including the improvement of medication adherence, attendance of medical appointments, and disease self‐management.^16^, ^17^ More specifically, using mobile phones to deliver short message service (SMS), or text messages, can impact behavior modifications. Text messaging allows for the delivery of individualized health communication and reinforcement.^18^ A systematic review of health promotion and behavior interventions found that personalized periodic reminders for modifying diet, activity, and weight are effective to encourage and reinforce healthy behaviors.^19^

Studies evaluating the effectiveness of using text messaging for the promotion of healthy weight loss behaviors and improvement of diabetes control in racial and ethnically diverse communities show promise as demonstrated in the feasibility of text messages.^20^, ^21^, ^22^ Wadden at al.^23^ commented that digitally delivered obesity programs expand treatment reach and lower costs. Although a recent systematic literature review on mHealth technology in historically underserved and minority populations in the United States failed to find an article on mHealth and obesity in this population,^24^ we found several references of weight loss in this population. A randomized control trial with 124 African American adults who were overweight or obese found that the group who participated in a 6‐month text messaging program in addition to the standard of care had an added weight loss of 3.5 kg, on average, than standard‐care control at 6 months.^25^ Additionally, a 12‐month effectiveness randomized control trial with socioeconomically disadvantaged patients with obesity and elevated cardiac risk found that a mobile app behavior change intervention plus physician counseling rendered a larger weight loss relative to usual primary care in a community health care system.^26^ A randomized control trial in Belgium comparing a conventional face‐to‐face weight loss program, a weight loss mobile app program, a combination of both, and a control found that while all interventions achieved weight loss from baseline, there was a trend of a greater number of participants in the combination group losing at least 5% of baseline weight compared to the mobile app group alone, suggesting that the combination of in‐person interaction and mobile technology might be more effective to reaching weight control.^27^

Thus, we sought to test the hypothesis that text‐messaging programs would be a useful and effective strategy to help socioeconomically disadvantaged adults in a safety‐net setting improve weight outcomes, hemoglobin A1c (HbA1c), and lipid levels compared to those in the medical group visit alone.

## 2 MATERIALS AND METHODS

The POWER Obesity Group Visit, which stands for Preventing Obesity With Eating Right, is an obesity medical group visit that was started in 2013 at the Martin Luther King, Jr. Outpatient Center (MLK OC). MLK OC is an inner‐city outpatient clinic in South Los Angeles that provides care for low‐income, mostly racial/ethnic minority participants that is supported by Los Angeles County Department of Health Services (LAC‐DHS). Referrals to the POWER clinic were mostly from primary care physicians at MLK OC, but also came from health care providers throughout LAC‐DHS. The POWER clinic was held on a weekly basis; the 1^st^, 3^rd^, and 5^th^ Mondays were for English‐speaking participants and the 2^nd^ and 4^th^ Mondays were for Spanish‐speaking participants. Each session began with a thirty‐minute Zumba exercise, followed by a live discussion on various topics led by an Endocrinologist (Theodore C. Friedman). Topics included obesity complications and prevention, with an emphasis on lifestyle changes. Additionally, Theodore C. Friedman encouraged open discussions to review participant challenges and personal experiences. Guest speakers were also invited to give brief talks to help address participants' main concerns and provide additional information. A dietitian also gave a lecture for about 45 min based on the Diabetes Prevention Program (DPP).^28^ The study was approved by the Charles R. Drew University IRB (CDU IRB # 13‐08‐2409) and all participants signed an informed consent to participate in the study.

We collected data from the patients in the POWER obesity program from October 2013 to May 2018. Starting in April 2015, patients in the POWER obesity program were given the opportunity to enroll in the texting programs offered by CareMessage^TM^, which focuses specifically on medically underserved populations. At the end of each POWER obesity clinical session, a 15‐min presentation was given to discuss and review the features of the texting program. Participants in cohort 1, the control group, attended the group visits only and did not receive any text messages. These include subjects who were in the POWER obesity program that came multiple times either before the texting program was instituted in April 2015 or those who chose not to participate in the texting program. Those in cohort 2 attended the group visits and participated in the 12‐week texting program during the period of April 2015 to May 2016, while those in cohort 3 enrolled in the 20‐week texting program during the period from May 2016 to March 2018.

The texting programs were available and made free of charge to all participants aside from standard text messaging charges. Participants chose whether they wanted to receive the text messages in English or Spanish. Those who enrolled in the 12‐week and 20**‐**week programs received three text messages per week. The texts included appointment reminders, health and wellness tips, and educational information related to care and disease management. The 12‐ and 20‐week programs allowed patients to set goals around exercise or nutrition. Furthermore, the 20‐week program included motivational, mental health, and stress management messages to help encourage healthy lifestyle changes. The 20‐week program allowed participants to choose from a broader set of health goals including, increase water consumption, exercise more often, cook healthier meals, or practice portion control. Participants were also provided additional information and resources to assist with mobile technology use, including the state program California Life Line, in which those eligible could obtain discounted cell phone services. In addition, research staff offered one‐on‐one sessions after the clinic for participants who needed help with texting. Participants were encouraged to attend the POWER group clinic regularly, as well as voice any issues or concerns they had regarding any aspect of the visit.

**FIGURE 1:**
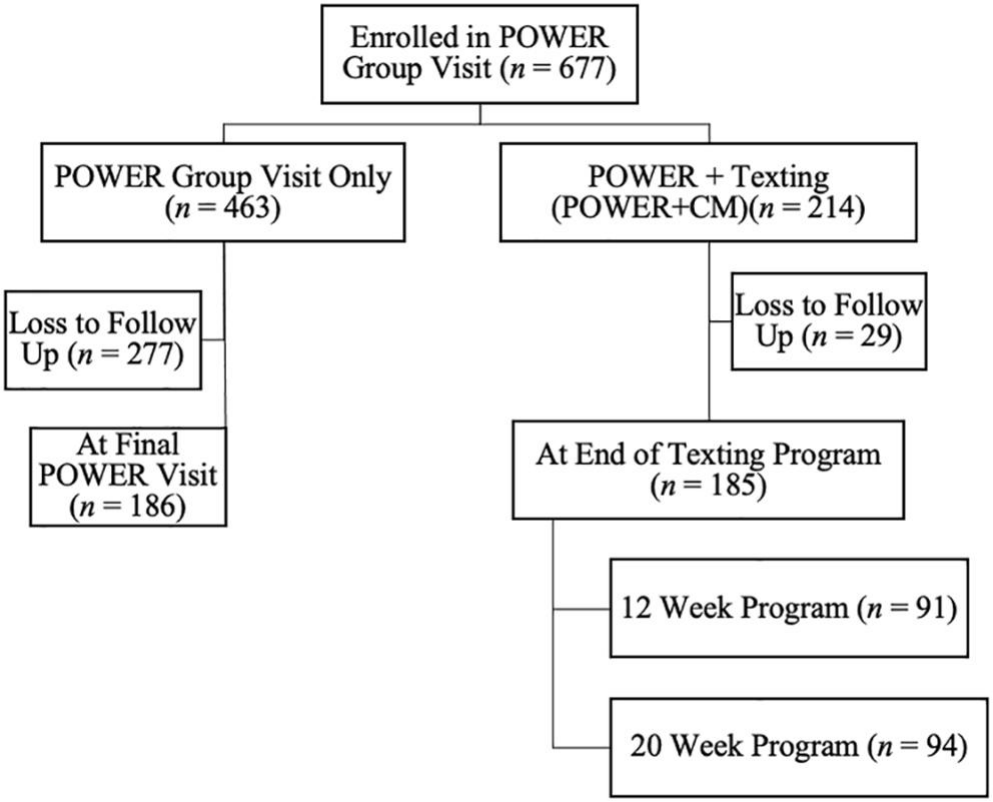
Diagram of participant enrollment starting October 2013

At each clinic visit, data was collected on each participant and entered into the study database. These variables included the participant's name, date of birth, medical record number, dates of attendance, blood sugar and lipid levels, medications, height, and weight. Figure 1 shows the number in each group and dropouts.

### 2.1 Power and statistical analysis

Following the suggestion from Leon et al.^29^ if we select a clinically meaningful effect of 3 pounds between text messaging intervention and group visit alone and assuming a standard deviation of 9 pounds then with 186 subjects in the group visit only arm and 185 individuals in the group visit plus text messaging arm, and with a 2‐tailed 5% significance level, there would be 89.3% power to detect a similar effect size (0.33 SD, i.e., Cohen's D).^30^ For the univariate analyses, the Wilcoxon rank‐sum test was used to compare quantitative outcomes between the two main study groups because of the uncertainty in the underlying probability distributions. For evaluating changes within group, the Wilcoxon signed‐rank test was used. In some instances when there were three groups involved (text messaging program status), the Kruskal–Wallis nonparametric analysis of variance was the test of choice and Dunn's test was used for evaluating individual intergroup differences in the event of three group significance. For qualitative (categorical) data, chi‐square tests for homogeneity were employed to compare proportions in each group. Spearman rank correlations (Pearson's correlation based on ranks) were used to evaluate the relationships between various outcomes and characteristics of the POWER program. The test of significance for this correlation was based on the appropriate t‐statistic. When there were missing values in time dependent results, the simple last‐value‐carried‐forward (LVCF) methodology was used to impute results. However, we performed a sensitivity analysis to determine whether imputation had any effect on the results by using only completed data as well.

## 3 RESULTS

### 3.1 Participant baseline characteristics

A total of 371 participants were included in the study and their baseline characteristics are shown in Table 1. The majority of participants were female (83.3%) with a mean age of 54.1 years (*SD* = 10.8) (age range 26–80 years) and Hispanic (51.2%). A total of 191 participants selected English as their preferred language while 180 participants preferred Spanish. Of those who attended at least two group clinic sessions, 369 participants had weight values, 269 had HbA1c values, and 277 participants had lipid values at baseline and after intervention. About half of the total participants (*n* = 186) participated in the POWER group visit only and the other half (*n* = 185) participated in the POWER group visit and the text messaging interventions. For those who enrolled in the texting program, 91 participants completed the 12‐week text messaging program, and 94 completed the 20‐week text messaging program.

**TABLE 1:** Participant characteristics

Characteristic	Control (*n* = 186)	Texting Intervention (*n* = 185)	Total (*n* = 371)	*p‐*value
Age, years (mean ± SD)	54.1 ± 10.5	54.1 ± 11.1	54.1 ± 10.8	0.98
Sex female, *n* (%)	154 (82.8)	155 (83.8)	309 (83.3)	0.80
Race/Ethnicity, *n* (%)				0.065
African American	75 (40.3)	97 (52.4)	172 (46.4)	
Hispanic	106 (57.0)	84 (45.4)	190 (51.2)	
Other (Asian/White)	5 (2.7)	4 (2.2)	9 (2.5)	
Preferred language, *n* (%)				0.015^*^
English	84 (45.2)	107 (57.8)	191 (51.5)	
Spanish	102 (54.8)	78 (42.2)	180 (48.5)	
Weight, kg (mean ± SD) (*n* = 371)	114.0 ± 26.5	112.3 ± 28.6	113.2 ± 27.6	0.55
BMI, kg/m^2^ (mean ± SD) (*n* = 371)	43.1 ± 9.5	43.0 ± 9.7	43.1 ± 9.6	0.89
HbA1C, % (mean ± SD) (*n* = 269)	7.0 ± 1.7	6.8 ± 1.7	6.9 ± 1.7	0.25
Lipids, mg/dL (mean ± SD) (*n* = 277)				
Cholesterol	186 ± 57	180 ± 41	183 ± 50	0.31
HDL	46 ± 12	49 ± 14	48 ± 13	0.02^*^
LDL	109 ± 53	111 ± 60	110 ± 57	0.70
Non‐HDL	141 ± 58	129 ± 39	135 ± 49	0.046^*^
Triglycerides	151 ± 96	156 ± 153	154 ± 127	0.74
Intervention program, *n* (%)				
12‐Week texting		91 (24.5)		
20‐Week texting		94 (25.3)		

*Note: p*‐values were calculated using or the quantitative data, the Wilcoxon rank‐sum test was used; for the qualitative data, the chi‐square test for homogeneity.

Abbreviations: BMI, body mass index; HbA1C, hemoglobin A1c; HDL, high‐density lipoprotein; LDL, low‐density lipoprotein; Non‐HDL, Non‐high‐density lipoprotein.

^*^ Difference between groups was significant *p* < 0.05.

When comparing the POWER group visit only (control group) and the POWER group visit plus texting programs, a larger number of participants in the texting intervention indicated English as their preferred language (*n* = 107 vs. *n* = 84), and more enrollees in the control group indicated Spanish as their preferred language (*n* = 102 vs. *n* = 78; *p* < 0.05, see Table 1). Aside from the significantly higher HDL levels in the POWER group visit plus texting program groups (49 ± 14 mg/dl vs. 46 ± 12 mg/dl, *p* < 0.05) group and the significantly higher non‐HDL levels in the POWER only group (141 ± 58 mg/dl vs. 129 ± 39 mg/dl, *p* < 0.05), there were no other significant differences in baseline clinical values between both groups.

### 3.2 Medical group visit attendance

Those enrolled in the 20‐week and 12‐week texting programs attended more group visit sessions than those enrolled in the POWER group only (*p* < 0.0001). There was no significant difference in the number of group visits attended between the 20‐week and 12‐week texting programs.

### 3.3 Weight loss

Both POWER and POWER + 20‐week texting groups had a significant reduction in weight (in kg) at their final group visit compared to their baseline (POWER, 114 ± 27 kg vs. 112 ± 26 kg, *p* < 0.001; POWER + 20‐week texting, 111 ± 28 kg vs. 109 ± 28 kg, *p* = 0.002), but not the 12‐week texting program (114 ± 29 kg vs. 113 ± 29 kg, *p* = 0.22) (Table 2). Between‐group analysis showed no significant difference between groups. At the final group visit, both the POWER only group (−1.9 ± 91 kg, *p* < 0.001) and the POWER + 20 weeks texting group (−1.5 ± 4.9 kg, *p* < 0.001) showed weight loss compared to baseline, while there was no significant weight loss in the POWER + 12‐weeks texting group (−0.7 ± 4.9  kg, *p* > 0.05) (Table 2). When both texting components were combined, weight loss was −1.10 ± 4.87 kg (*p* = 0.48 vs. POWER alone group). Percentage weight loss also occurred in the POWER only group (−1.2 ± 4.3%, *p* < 0.001), as well as the 20‐week (−1.1 ± 4.7%, *p* < 0.001), but not the 12‐week texting program (−0.6 ± 3.8%, *p* = 0.13) (Table 2). Between‐group analysis showed no significant difference for either weight loss in kg or% weight loss between groups when the two texting programs were examined separately or together. Both weight loss in kg and % weight loss were positively correlated with number of visits [(*r*_*s*_) = 0.12, *p* = 0.022] and [(*r*_*s*_) = 0.12, *p* = 0.018], respectively. When examining only those enrolled in a texting program, there was a significant negative correlation between the number of messages participants replied to and the amount of weight loss for the 12‐week texting program, meaning increased messages was associated with a decrease in weight (*r*_*s*_ = −0.22, *p* < 0.05), but not for the 20‐week texting program.

**TABLE 2:** Changes in clinical measures by texting enrollment

	Control (*n* = 186)			Texting Intervention (*n* = 185)						
				12‐Week program (*n* = 91)			20‐Week program (*n* = 94)			
Variable	Baseline	Final	*p*	Baseline	Final	*p*	Baseline	Final	*p*	*p‐*value between groups
Body weight, kg	114 (27)	112 (26)	<0.001*	114 (29)	113 (29)	0.22	111 (28)	109 (28)	0.002*	0.25
Weight loss, kg		−1.9 (9.1)	<0.001*		−0.7 (4.9)	0.22		−1.5 (4.9)	0.002	0.25
Weight loss, %		−1.2 (4.3)	<0.001*		−0.6 (3.8)	0.13		−1.1 (4.7)	0.003^*^	0.30
HbA1c, %	6.8 (1.7)	6.7 (1.6)	<0.001*	6.8 (1.5)	6.7 (1.4)	0.26	6.6 (1.8)	6.3 (1.4)	0.007^*^	0.16
Cholesterol, mg/dL	186 (53)	179 (44)	0.17	178 (44)	179 (47)	0.97	183 (420	178 (38)	0.15	0.54
HDL, mg/dL	46 (11)	46 (12)	0.99	50 (13)	48 (12)	0.12	50 (14)	50 (13)	0.78	0.36
LDL, mg/dL	109 (49)	104 (37)	0.36	105 (49)	103 (51)	0.37	115 (63)	105 (44)	0.24	0.83
Non‐HDL, mg/dL	140 (54)	134 (40)	0.28	124 (43)	124 (48)	0.80	132 (38)	129 (38)	0.30	0.65
Triglycerides, mg/dL	152 (98)	147 (86)	0.29	152 (103)	153 (113)	0.35	151 (170)	134 (90)	0.89	0.93

*Note: p*‐values for within group comparisons were calculated using the Wilcoxon signed‐rank test. *p*‐values for the between group comparisons used the Wilcoxon rank‐sum test.

Abbreviations: HbA1c = hemoglobin A1c; HDL = high‐density lipoprotein, LDL = low‐density lipoprotein, Non‐HDL= Non‐high‐density lipoprotein.

^*^ Difference within group was significant *p* < 0.01.

### 3.4 Hemoglobin A1c

Participants in both the POWER and POWER + 20‐week texting groups experienced a significant reduction in HbA1c percentage from baseline (POWER, 6.8 ± 1.7% vs. 6.7 ± 1.6% *p* < 0.001; POWER + 20‐week texting program, 6.6 ± 1.8% vs. 6.3 ± 1.4%, *p* < 0.01), but not the POWER + 12‐week texting program, 6.8 ± 1.5% versus 6.7 ± 1.4%, *p* = 0.26 with no significant difference between groups.

### 3.5 Lipids

When examining lipids, there was no difference in cholesterol, LDL, non‐HDL, or triglycerides between baseline and final measurements in the control group, the 12‐week texting, and 20‐week texting groups (Table 2). There was no difference between groups.

## 4 DISCUSSION

Our findings demonstrate that a medical group visit program led to weight loss and improvement in HbA1c levels in racial and ethnic minorities with obesity. We are writing up a manuscript that describes the changes in weight and HbA1c in POWER obesity participants compared to historic controls (Pulido et al., in preparation). Although the text messaging component led to modest weight changes, it did not enhance the weight loss and glycemic improvement from the medical group visit. Our finding that the text messaging component did not add to weight loss and glycemic improvement was unexpected. However, it is noteworthy that prior studies on text messaging in underserved and minority populations that found weight loss were in comparison to usual care^25^, ^26^ and did not examine the supplementary effect of text messaging in addition to a weight loss intervention. The lack of an additive effect may be due to (1) participants were already motivated and educated by the group visits and the text messaging did not add to the effect, (2) the participants were not comfortable to send and receive text messages, (3) the text messaging did not cover the breadth of cultures in this population, or (4) the study was underpowered. The second and third explanations are unlikely as CareMessage^TM^ specializes in delivering culturally appropriate messages to patients with low technology literacy. The study had 89.3% power to detect a difference between text messaging intervention and group visit alone, so the study is unlikely to be under‐powered. Further reasons for lack of effect are listed under the limitations section below.

Overall, attending the POWER group visit was beneficial for both those who enrolled in text messaging and those who did not. Findings from the current study are consistent with previous pilot interventions that use shared medical appointments as cost‐efficient and effective alternatives for weight loss.^31^ Delivering diet and physical activity interventions in group settings (not shared medical appointments) has shown to be effective in promoting weight loss at 12 months or greater.^32^, ^33^ A recent large study in rural primary care clinics found that in‐clinic group visits but not telephone‐based group visits, compared with in‐clinic individual visits, resulted in statistically significantly but modest weight loss at 24 months.^34^ In our study, those who enrolled in the 20‐week texting program and those who did not enroll in any texting program had significant weight loss.

Participants, on average, had a reduction in HbA1c values, with the POWER + 20‐week messaging group experiencing a 0.3% reduction resulting in HbA1c values below 6.5%, which have been associated with decreased risk of mortality due to CVD and cancer.^35^ A previous study also showed that patients in a text message intervention achieved greater reduction in HbA1c than those in usual care.^21^

Several strengths of the study are worth noting. The study had a large sample size that was adequately powered to detect an effect of the intervention. The medical group visit combined peer support with a lifestyle modification program. Social support has been associated with greater weight loss and maintenance in lifestyle interventions.^36^ Texts provide support in real‐time and real‐world setting. Those enrolled in the texting program received appointment reminders, which has been associated with greater adherence to lifestyle studies in ethnic/racial minorities.^37^ Increased adherence is important when evaluating cost of no‐show appointments, estimated to be $196 per patient in 2008.^38^ Further, this study addresses the barriers to weight loss of physical activity companionship and food advice reported by Spanish‐speaking women in previous studies.^39^

Study findings should be interpreted in the context of several limitations. First, all participants in the study had to attend the group visit at least twice. Thus, 29 consented participants who did not return for a follow‐up visit were exempt from study. Further, group visits were scheduled at 1300 every Monday, which may have led to less individuals with a set work schedule being able to participate. Study participants were not randomized into the texting groups or the POWER obesity control, so self‐selection may have been a factor, although this would be more likely to show a difference in the text messaging programs that did not occur. The texting groups and the control were done at different periods of time, so some effects may be due to the timing and not to the effect of the group.

Limitations with the texting program should be noted. First, standard charges for texts from cell phone carriers may have limited some participants from entering and did not permit us to randomize which participants enrolled in the texting program. This may have led to a selection bias in those who enrolled in the text messaging programs. Moreover, it is unknown how much of the message content was read, comprehended, and applied. Notably, some of our participants reported having difficulty reading the messages due to the small font. Further, there were troubleshooting issues including lack of notification of cell phone number change, and inadvertently stopping the texting program. A short survey administered to those who did not enroll in the texting program assessed reasons for declining participation. Reasons to decline participation included security concerns due to lack of encryption, severe physical disabilities, texting charges, and lack of fluency in text language. One‐on‐one sessions were offered to learn how to text before enrolling in the texting program, but it is unknown whether those lessons were enough for participants to gain texting competency.

In summary, group visits and group visit plus text messaging led to weight loss and improvement in HbA1c in African Americans and Hispanics with obesity in a low‐income urban area with the effect being due to the group visit without the added benefit of a text messaging program. Our findings support the recommendation of Bennett and colleagues^26^ that digital obesity treatments including text messaging should not be used as replacements for individual or group interventions, but could be used within comprehensive obesity treatment programs to deliver educational materials, offer tailored feedback and facilitate encounters with providers. Further studies are needed to explore the applicability of text messaging with lifestyle modification programs within a safety‐net setting.

## CONFLICT OF INTEREST

The authors have no conflict of interest.

## AUTHOR CONTRIBUTIONS

Study Concept and Design: Perla Saldivar, Valerie Mira, Petra Duran, Georgina Ang, Nina Parikh, Theodore C. Friedman, Data Analysis and Interpretation: Perla Saldivar, Valerie Mira, Petra Duran, Martin L. Lee, Theodore C. Friedman, Manuscript Preparation: Perla Saldivar, Valerie Mira, Nina Parikh, Christina Moldovan, Petra Duran, Georgina Ang, Martin L. Lee, Theodore C. Friedman.
